# Early intervention with azelastine nasal spray may reduce viral load in SARS-CoV-2 infected patients

**DOI:** 10.1038/s41598-023-32546-z

**Published:** 2023-04-26

**Authors:** Jens Peter Klussmann, Maria Grosheva, Peter Meiser, Clara Lehmann, Eszter Nagy, Valéria Szijártó, Gábor Nagy, Robert Konrat, Michael Flegel, Frank Holzer, Dorothea Groß, Charlotte Steinmetz, Barbara Scherer, Henning Gruell, Maike Schlotz, Florian Klein, Paula Aguiar de Aragão, Henning Morr, Helal Al Saleh, Andreas Bilstein, Belisa Russo, Susanne Müller-Scholtz, Cengizhan Acikel, Hacer Sahin, Nina Werkhäuser, Silke Allekotte, Ralph Mösges

**Affiliations:** 1 Center for Molecular Medicine Cologne (CMMC), Faculty of Medicine and University Hospital, University of Cologne, Kerpener Str. 62, 50937 Cologne, Germany; 2 Medical Faculty, Department of Otorhinolaryngology, Head and Neck Surgery, University of Cologne, Kerpener Str. 62, 50937 Cologne, Germany; 3 URSAPHARM Arzneimittel GmbH, Industriestraße 35, 66129 Saarbruecken, Germany; 4 Department I of Internal Medicine, Division of Infectious Diseases, University of Cologne, Kerpener Str. 62, 50937 Cologne, Germany; 5 German Center for Infection Research (DZIF) Location Bonn-Cologne, Kerpener Str. 62, 50937 Cologne, Germany; 6 CEBINA GmbH, Karl-Farkas-Gasse 22, 1030 Vienna, Austria; 7 Department of Structural and Computational Biology, Max F. Perutz Laboratories, University of Vienna, Dr.-Bohr-Gasse 9, 1030 Vienna, Austria; 8 Laboratory of Experimental Immunology, Institute of Virology, Faculty of Medicine and University Hospital, University of Cologne, Kerpener Str. 62, 50937 Cologne, Germany; 9 Ursatec GmbH, Marpinger Weg 4, 66636 Tholey, Germany; 10 ClinCompetence Cologne GmbH, Theodor-Heuss-Ring 14, 50668 Cologne, Germany; 11 Institute of Medical Statistics and Computational Biology (IMSB), Faculty of Medicine, University of Cologne, Kerpener Str. 62, 50937 Cologne, Germany

**Keywords:** Randomized controlled trials, Respiratory tract diseases, Viral infection

## Abstract

With the changing epidemiology of COVID-19 and its impact on our daily lives, there is still an unmet need of COVID-19 therapies treating early infections to prevent progression. The current study was a randomized, parallel, double-blind, placebo-controlled trial. Ninety SARS-CoV-2 positive patients were randomized into 3 groups receiving placebo, 0.02% or 0.1% azelastine nasal spray for 11 days, during which viral loads were assessed by quantitative PCR. Investigators assessed patients’ status throughout the trial including safety follow-ups (days 16 and 60). Symptoms were documented in patient diaries. Initial viral loads were log_10_ 6.85 ± 1.31 (mean ± SD) copies/mL (ORF 1a/b gene). After treatment, virus load was reduced in all groups (*p* < 0.0001) but was greater in the 0.1% group compared to placebo (*p* = 0.007). In a subset of patients (initial Ct < 25) viral load was strongly reduced on day 4 in the 0.1% group compared to placebo (*p* = 0.005). Negative PCR results appeared earlier and more frequently in the azelastine treated groups: being 18.52% and 21.43% in the 0.1% and 0.02% groups, respectively, compared to 0% for placebo on day 8. Comparable numbers of adverse events occurred in all treatment groups with no safety concerns. The shown effects of azelastine nasal spray may thus be suggestive of azelastine’s potential as an antiviral treatment.

**Trial registration:** The study was registered in the German Clinical Trial Register (DRKS-ID: DRKS00024520; Date of Registration in DRKS: 12/02/2021). EudraCT number: 2020-005544-34.

## Introduction

Since viral levels during early infection with Severe Acute Respiratory Syndrome Coronavirus 2 (SARS-CoV-2) tend to be highest in the nose and nasopharynx^1^, a nasal spray with an active substance inhibiting virus entry and replication may stop or delay the progression of the disease to the lower respiratory system and reduce the transmission to uninfected individuals.

Azelastine hydrochloride nasal spray is an approved medicinal product currently available at a concentration of 0.1% w/v to treat allergic rhinitis. The active substance (azelastine hydrochloride) is a histamine-1 receptor antagonist, which shows anti-inflammatory effects via mast cell stabilization and inhibition of leukotriene and pro-inflammatory cytokine production^2–4^.

Since the start of the Coronavirus Disease 2019 (COVID-19) pandemic, several independent research groups revealed azelastine’s potential as a promising candidate for drug repurposing to reduce SARS-CoV-2 viral load and infection rates^5–10^. In an in vitro screening of 1,800 approved drugs by use of a SARS-CoV-2-S pseudovirus entry inhibitor model, 15 drugs were identified as active inhibitors, but only seven of these drugs were identified as active against SARS-CoV-2, three of which were anti-histamines: clemastine, trimeprazine and azelastine hydrochloride^5^. Reznikov et al. analyzed 219,000 medical records in a retrospective data base survey study and demonstrated that azelastine showed the highest association between prior usage among these antihistamines and SARS-CoV-2 negative test results in patients above the age of 60 (OR: 2.43; 95% CI: 1.47–4.02). Antiviral activity was subsequently verified in cell culture. Moreover, this group showed that azelastine has the potential to inhibit SARS-CoV-2 cell entry by binding to the angiotensin-converting enzyme 2 (ACE2) receptor and to inhibit intracellular virus replication through binding to the sigma-1 receptor^6^. Furthermore, three independent groups predicted interaction of azelastine hydrochloride with the main protease of SARS-CoV-2: main protease (Mpro) or 3C-like cysteine protease (3CLpro)^7–9^. Ghahremanpour et al. also provided experimental evidence for the inhibition of the enzyme in a kinetic activity assay^7^.

By application of a novel computational approach based on Shannon entropy homology, Konrat et al. identified azelastine as an anti-viral candidate and demonstrated pronounced anti-SARS-CoV-2 activity in vitro^10^. Antiviral efficacy was observed at an EC_50_ of ~ 6 µM, which is an approximately 400-fold lower concentration compared to commercially available azelastine nasal sprays. In a highly relevant and translational in vitro model using reconstituted human nasal tissue, a fivefold diluted commercially available azelastine nasal spray solution inhibited viral replication almost completely within 72 h after SARS-CoV-2 infection^10^.

The aim of our study was to support the preclinical evidence for azelastine’s antiviral activity in patients tested positive for SARS-CoV-2. The study was termed CARVIN (referring to **C**OVID-19: **A**zelastine nasal spray **R**educes **V**irus-load **I**n **N**asal swabs).

### Ethics declarations

Ethics approval was granted by the Ethics Committee of the Faculty of Medicine of Cologne University on the 10^th^ of February 2021. Approval of the study by the German Federal Institute for Drugs and Medical Devices (BfArM) was given on 3rd February 2021.

Informed consent was obtained from all participants prior to involvement in the study.

## Patients and methods

All methods were carried out in accordance with relevant guidelines, and the principles of Good Clinical Practice and the Declaration of Helsinki were adhered to.

### Study setting

This trial was conducted at the Department of Otorhinolaryngology, Head and Neck Surgery of the Faculty of Medicine of the University of Cologne, Germany. Outpatients visiting Corona test centres were informed about the possibility of participating in the trial. Patients aged 18 to 60 years were eligible to participate if tested positive for SARS-CoV-2 in a Corona test centre by PCR test within 48 h prior to inclusion and had to quarantine at home due to instructions of the local health authority. A complete list of inclusion and exclusion criteria is presented in Table 1. Patients were visited and tested at home on regular basis by the investigators, physicians specialised in otorhinolaryngology, medical hygiene, or general medicine.

**Table 1:** Inclusion and exclusion criteria for study participation.

Inclusion criteria	Exclusion criteria
Legally competent patients capable of given informed consent	Hospitalization
Aged 18–60 years old	Simultaneous participation in other clinical trial or previous participation within 30 days before inclusion
Positive PCR test for SARS-CoV-2 (nasal swab taken no longer than 48 h)	SARS-CoV-2 test older than 48 h
Females: Non-pregnant, non-lactating, with adequate contraception or unable to bear children	Relationship or dependence with the Sponsor, CRO and/or Investigator
	Risk of serious course of the disease (e.g. insulin-dependent diabetic patients, use of antihypertensive drugs)
	Inability to understand instructions/study documents
	Inability to administer the nasal spray
	Vulnerable patients: detained or committed to institutions by law court or legal authorities
	Females: pregnant, lactating, or of child-bearing potential and not using an adequate contraceptive method
	Concurrent anti-histamine therapy
	Concurrent nasal spray
	Contraindication for the use of azelastine (incl. hypersensitivity to the active substance or other ingredients)

### Study design

This was a prospective, randomized, double-blind, placebo-controlled dose-finding proof-of-concept study, in which azelastine nasal spray was used in 2 doses: the commercially available concentration of 0.1% and a fivefold lower concentration of 0.02%. After having given informed consent, patients tested positively for SARS-CoV-2 were examined to assess eligibility according to inclusion/non-inclusion criteria and subsequently randomized to one of the three study groups. The first administration of the nasal spray was carried out in the presence of the investigator; products were subsequently self-administered for 11 days (treatment phase). During the treatment phase, 7 visits (V1–V7) took place on days 1, 2, 3, 4, 5, 8 and 11. Samples of day 1 represent pre-treatment baseline samples. During visits, nasopharyngeal swabs were taken for quantitative PCR measurements, and investigators assessed the patient status in accordance with the WHO clinical progression scale^11^. Short intervals of swab collection time points, particularly during early days of infection, and high number of PCR tests aimed to monitor SARS-CoV-2 viral loads as closely as possible, considering that only limited knowledge regarding details of viral clearance was publicly available at the time of the study development. Additionally, safety follow-ups were performed at 2 time-points. On day 16, an on-site visit (V8) for female patients was conducted to perform a urine pregnancy test and to assess the safety of the therapy. For male patients, the assessment was done via phone call. A final safety follow-up and assessment of the patient status (WHO scale) by phone call was done on day 60 (V9) for all patients.

Patient reported outcomes were documented by patient diaries and questionnaires. Therefore, during the treatment phase, patients were required to document the severity of their COVID-19 related symptoms in an electronic diary on a daily basis. On days 1, 5, 8 and 11, patients completed the standardized SF-36 questionnaire of quality of life. A summary of study activities is displayed in Table 2.

**Table 2:** Study flow chart.

	Treatment phase												Follow-up
Schedule (day)	1	2	3	4	5	6	7	8	9	10	11	16	60
Study visits	V1	V2	V3	V4	V5			V6			V7	V8	V9
Contact Study hotline	X												
Informed consent	X												
Inclusion & non-inclusion criteria	X												
Demographic data	X												
Temperature measurement	X	X	X	X	X			X			X		
Urine pregnancy test	X											X	
Oxygen saturation of the blood	X		X		X			X			X		
Sampling naso-pharyngeal swabs	X	X	X	X	X			X			X		
Quantitative PCR measurement	X	X	X	X	X			X			X		
Assessment of patient status	X	X	X	X	X			X			X		X
Safety assessment (Patient’s AE profile)	X	X	X	X	X			X			X	X	X
Documentation of symptoms (patient)	X----------------------------------------------------------------------------------------------------------------------------------------------------------------------------------------------------------------------------------------X												
Study drug administration	X----------------------------------------------------------------------------------------------------------------------------------------------------------------------------------------------------------------------------------------X												
SF-36 QoL	X				X			X			X		
Final assessment											X		

### Randomization and masking

Assignment of the treatment with the investigational medicinal product in the different doses vs. placebo to each treatment number was performed in a centrally conducted, computer-generated 1:1:1 randomization procedure. Treatment kits were manufactured by URSAPHARM Arzneimittel GmbH, Saarbruecken, Germany, according to the randomization list (as sequentially numbered containers). Patients were assigned a treatment number in an ascending mode according to their chronological order of inclusion. Investigators and trial participants were masked to the treatment as investigational medicinal products were identical in appearance.

### Intervention and comparator

The trial medication (placebo nasal spray, 0.02% azelastine nasal spray or 0.1% azelastine nasal spray (the latter being identically composed as the commercial anti-allergic product Pollival^®^) was manufactured at URSAPHARM Arzneimittel GmbH, Saarbruecken, Germany). All nasal sprays were composed of hypromellose, disodium edetate, citric acid, disodium phosphate dodecahydrate, sodium chloride and purified water. Additionally, 0.02% azelastine nasal spray and 0.1% azelastine nasal spray were formulated by the addition of 0.2 mg/mL or 1 mg/mL azelastine hydrochloride, respectively. One puff of the respective nasal spray was applied per nostril, 3 times a day (morning, midday, evening).

### Nasopharyngeal swabs

Nasopharyngeal swabs were obtained by investigators using nylon-flocked swabs (Biocomma; SW01E, flexible minitip, Biocomma, Shenzen, China). Following sampling, swabs were placed into 3 mL Virus Transport Medium (VTM, Biocomma) and delivered to the laboratory as quickly as possible. If delivery took place within 24 h after sampling, samples were to be stored at < 25 °C, if storage period was greater than 24 h (e.g., on Sundays), samples had to be stored and shipped at 2–8 °C. Samples were processed on the day of receipt at the central processing laboratory (Institute of Virology, University Hospital Cologne, Cologne, Germany) by vortexing and aliquoting the viral transport medium and stored at − 80 °C until analysis.

### Quantitative PCR

SARS-CoV-2 RNA levels in nasopharyngeal swabs were determined by quantitative RT-PCR using the cobas^®^ SARS-CoV-2 Test on the cobas^®^ 6800 system (Roche Diagnostic, Mannheim, Germany). For quantification of SARS-CoV-2-RNA in copies/mL, a standard curve derived from a dilution series of a SARS-CoV-2 cell culture isolate in VTM and adjusted to Ct values obtained from two samples with defined SARS-CoV-2-RNA copy numbers (10^6^ and 10^5^ copies/mL; INSTAND e.V., Duesseldorf, Germany) was used. For calibration purposes of quantitative assessments, reference samples were included with each PCR run. The dual-target RT-PCR independently targets the ORF1a/b and the sarbecovirus E genes, and assays were considered positive if at least one target returned a positive result (Ct values reflecting an inverse relationship with viral load). Of note, in vitro tests carried out prior to the current study did not indicate any interaction between the study products and the PCR reaction (see supplementary PCR data). For data analysis, negative PCR results were replaced with the Ct value 45 and the cp/mL value 1, respectively. Information on individual variants was obtained through the original laboratory reports, when available. Detection of the alpha (B.1.1.7) variant was based on single nucleotide polymorphism analysis for SARS-CoV-2 spike gene mutation N501Y and deletion H69/V70.

### Patient reported outcomes

Patients had to daily document their COVID-19 specific symptoms in an electronic patient diary. Those parameters were based on the COVID-19 symptoms published by the Robert Koch Institute (https://www.rki.de) at the time of the study. Symptoms were evaluated on a 5-point scale from 1 = symptom absent or present very weakly to 5 = symptom present very strongly: anosmia, ageusia, cough, sore throat, shortness of breath, coryza, general weakness, headache, aching limb, loss of appetite, pneumonia, nausea, abdominal pain, vomiting, diarrhea, conjunctivitis, rash, lymph node swelling, apathy, somnolence. In addition, presence or absence of fever (≥ 38.0 °C) was documented daily (0 = no fever, 3 = fever). Symptoms were analyzed as single symptom scores, and as the total symptom score (TSS) reflecting the sum of all 20 single symptoms and presence/absence of fever (reaching a minimum value of 20 and maximum value of 103).

In addition, patient's quality of life was evaluated by the SF-36 questionnaire, covering 36 items divided into the 8 quality of life domains ‘physical functioning’; ‘role limitations due to physical health’, ‘role limitations due to emotional problems’, ‘energy/fatigue’, ‘emotional well-being’, ‘social functioning’, ‘pain’, and ‘general health’^12^.

At the end of the study, patients and investigators assessed the overall tolerability and efficacy of the treatment as ‘very good (3)’, ‘good (2)’, ‘moderate (1)’ or ‘poor (0)’.

### Patient status determination

The patient status was assessed at V1–V7 and at V9 by the investigators with a 11-category ordinal score proposed by the WHO^11^. In addition, investigators measured body temperature during V1–V7 and oxygen saturation of the blood (using a finger pulse oximeter) on V1, V3, and V5, V6 and V7.

### Endpoints and objectives

The primary endpoint of the CARVIN study was the assessment of virus load kinetics of SARS-CoV-2 by determining the presence and amount of viral carriage via PCR. Applied treatment regimens aimed to explore differences regarding viral carriage upon treatment with azelastine compared to placebo. Secondary endpoints included the assessment of symptoms, patient status (using a 11-category ordinal score as proposed by the WHO^11^), body temperature and blood oxygen saturation, quality of life (reported in the SF-36 generic quality of life questionnaires) and safety (adverse events, including worsening of patient status/symptoms) over time.

### Statistical analysis

The sample size calculation was based on the expected reduction of virus load during the treatment considering 3 treatment arms. It was assumed that all treatment groups present identical baseline virus load at enrolment with a mean value of 5.5 log_10_ copies/mL ± 3 SD^13,14^. Since azelastine has been shown to inhibit viral replication by 99.9% in Vero E6 cell culture and in reconstituted human nasal tissue cultures, it was assumed that a reduction of 3-log in virus load would be seen within 3 days in actively treated patients, while no effect on virus load reduction would be seen in placebo treated patients. Assuming a pooled standard deviation of σ = 3 units, a two-sided α = 0.05 and a power of 90%, a sample size of 23 patients per treatment group was calculated. Anticipating a drop-out rate of 20%, the aim was to randomize 90 patients in total (30 patients per treatment group) to result in 23 patients per treatment group completing the study and being eligible for analysis.

Data was analysed primarily exploratively; there was no formal testing of a given hypothesis. Analyses were done on the entire data set (ITT) as well as on a subset population with high viral load defined by baseline Ct values below 25 (Ct < 25). Both descriptive and exploratory statistics were performed. Subgroups were analysed exploratorily (e.g., subgroups regarding gender, age, symptom severity, etc.).

Continuous data were described by statistical estimates (mean, standard deviation, median, minimum, and maximum values). Categorical data were described by absolute frequencies and percentage of valid cases. Ct values reported as “negative” were replaced with the value 45, and respective cp/mL values with the value 1, and cp/mL values < 2116 (ORF 1a/b gene) and cp/mL values < 1950 (E gene) were replaced with the value 1.

Study endpoints were presented by descriptive statistics, aiming to compare the course of viral load between the three treatment groups. Whereas PCR data of individual days served for daily comparisons between treatment groups, the area under the curve (AUC) value was used for the evaluation of the overall development of viral kinetics. While comparison of categorial variables between groups were performed by Chi square testing, continuous variables were compared using ANCOVA with the factors baseline, visit, and treatment group. All tests were performed two-sided and the type 1 error (α) was set to 5%. Three-group comparisons were analysed with Kruskal–Wallis test. For pairwise comparisons between treatment groups, Mann Whitney *U* test was performed, and significance levels were adjusted to *p* < 0.0167 based on the Bonferroni correction. Kaplan–Meier survival analyses with log-rank test were performed to display the occurrence of negative PCR test results upon treatment. To evaluate the total load during the study, AUC was calculated using a linear equation.

## Results

Preliminary results of the current study have been published as preprint^15^. It should be noted that the SARS-CoV-2 alpha variant (B.1.1.7) was the dominant variant in Germany during the enrolment phase of the current study^16^.

### Trial population

**Figure 1:**
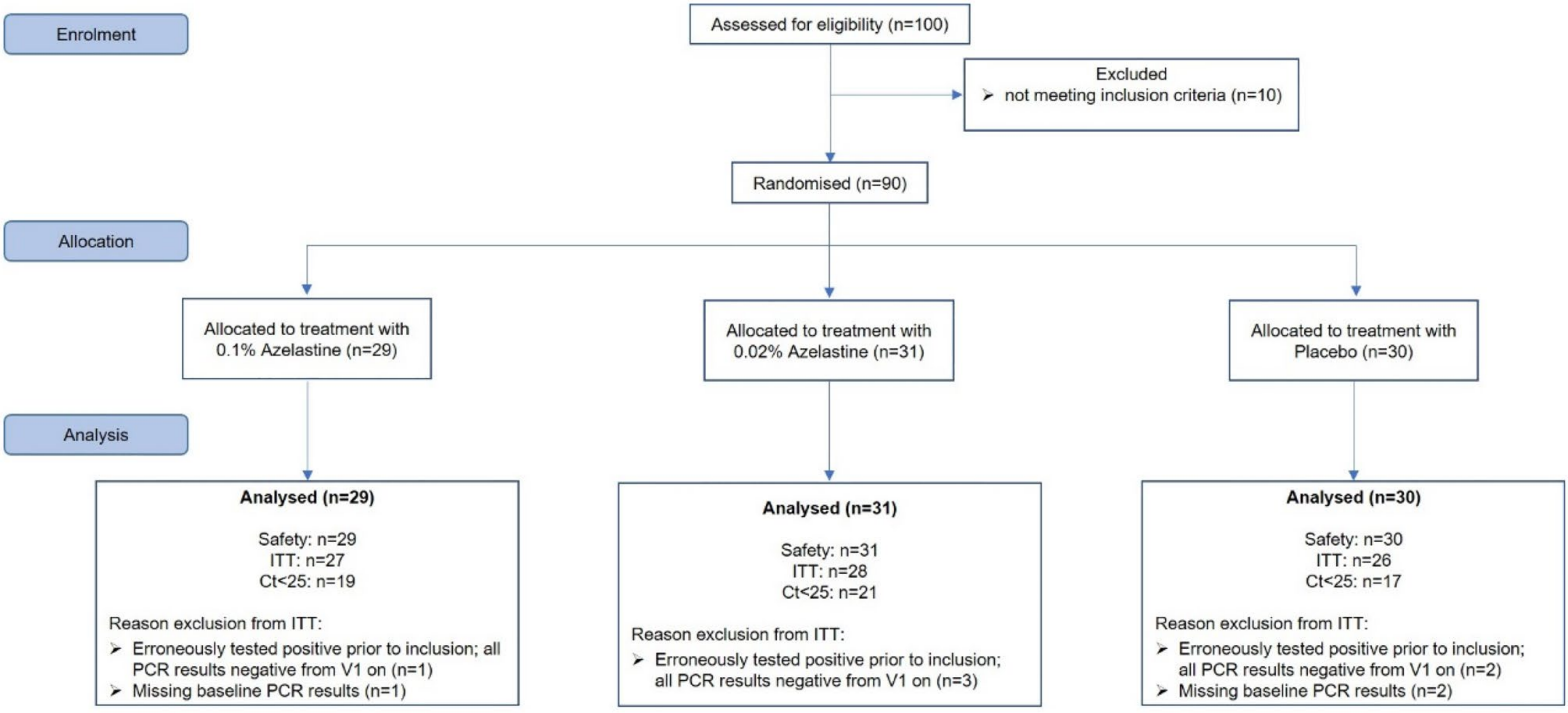
Patient disposition. ITT—intention to treat. Ct—cycle threshold.

90 patients were recruited between 09/03/2021 and 28/04/2021, constituting the safety analysis set. Of those, 81 patients belonged to the Intention-To-Treat (ITT) population, comprising randomised patients meeting the key eligibility criteria and having evaluable viral load data on day 1 (baseline) and on day 11 (end of treatment). Of those, 27 patients belonged to the 0.1% azelastine group, 28 patients to the 0.02% azelastine group and 26 patients to the placebo group (Fig. 1). The Ct < 25 group consisted of 19 patients in the 0.1% azelastine group, 21 patients in the 0.02% azelastine group and of 17 patients in the placebo group (Fig. 1).

### Demographics and baseline characteristics

Overall, no significant differences were observed between treatment groups regarding gender, age and body mass index (bmi, supplementary Table S1). 48.9% (*n* = 44) of the safety analysis set was male, and the average age was 35.67 ± 12.94 years. The mean bmi of participants was 24.91 ± 5.27. Small differences were found with regard to age and bmi, which were both slightly higher in the azelastine 0.1% group (supplementary Table S1).

### Kinetics of viral load

Overall, data of the primary outcome did not show a normal distribution (Shapiro–Wilk test, *p* < 0.05). Therefore, the primary analysis for the viral loads was conducted non-parametrically. For clarity reason, only cp/mL values of the ORF 1a/b gene are shown in the main text of the manuscript. As a sensitivity analysis based on the SARS-CoV-2 E gene PCR tended to show overall the same effects, PCR results of the E gene are shown in the supplementary material (supplementary Table S3 and S4).

The median/mean viral load value (ORF 1a/b gene) of the ITT analysis set at enrolment was log_10_ 7.23/6.85 ± 1.31 cp/mL (approximately 7 million viral copies per mL, the highest values being ~ 540 million cp/mL). Data on virus variants was available for 59 patients and 54 (92%) of those carried the alpha (B.1.1.7) variant.

**Figure 2:**
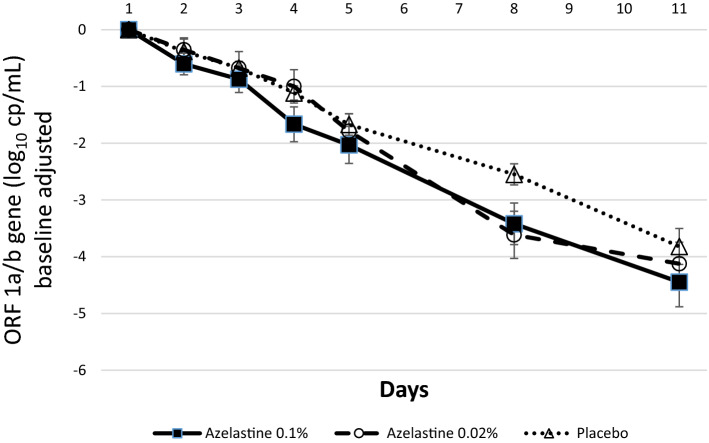
Absolute changes in viral copy numbers (log_10_ cp/mL) from baseline (day 1) over time based on the ORF 1a/b gene (ITT analysis set).

As expected, a continuous decrease in the mean virus load was observed in all study groups during the 11 treatment days. The reduction of virus load (reflected by decreases of ORF 1a/b gene copy numbers) from baseline to the end of treatment (day 11) was log_10_ 4.45 ± 2.26 in the 0.1% azelastine group, log_10_ 4.12 ± 2.01 in the 0.02% azelastine and log_10_ 3.82 ± 1.61 in the placebo group (Fig. 2 and supplementary Table S2). The reduction in virus load over the entire treatment period was clinically meaningful for all three groups (*p* < 0.0001 for both genes).

**Figure 3:**
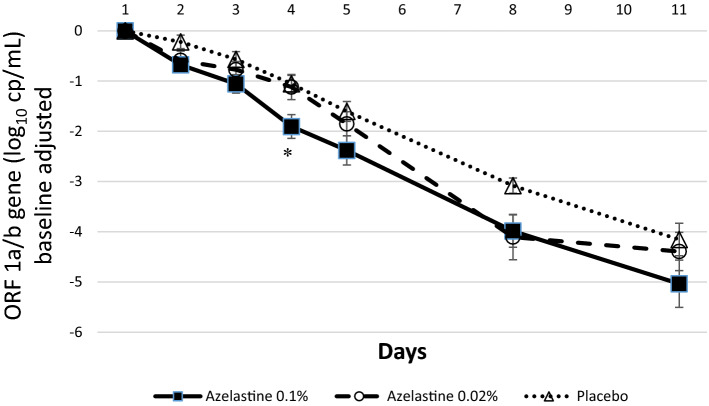
Absolute changes in viral copy numbers (log_10_ cp/ml) from baseline (day 1) over time based on the ORF 1a/b gene (Ct < 25 analysis set). *p = 0.005 comparing the decrease of viral load on day 4 in the 0.1% azelastine group (log_10_ 1.90 ± 1.03) compared to placebo (log_10_ 1.05 ± 0.70; *p* = 0.005).

Within the subgroup of patients with baseline Ct values below 25, a similar progression of viral load data was observed (Fig. 3). The viral load reduction of the ORF 1a/b gene from baseline to day 11 was log_10_ 5.04 ± 2.05 in the 0.1% azelastine group, log_10_ 4.39 ± 1.74 in the 0.02% azelastine and log_10_ 4.15 ± 1.34 in the placebo group. Of note, the decrease of viral load on day 4 was significantly greater in the 0.1% azelastine group (decrease by log_10_ 1.90 ± 1.03) compared to placebo (decrease by log_10_ 1.05 ± 0.70).

**Figure 4:**
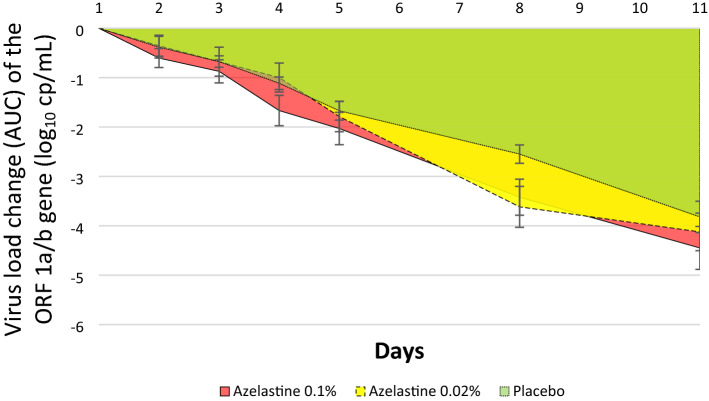
Area under the curve (AUC) reflecting changes in viral copy numbers (log_10_ cp/mL) from baseline (day 1) over time (until day 11) based on the ORF 1a/b gene (ITT analysis set). The overall AUC of the Azelastine 0.1% group (red area) was significantly greater than that of placebo (green area), *p* = 0.007.

Evaluation of AUC values (reflecting baseline adjusted decreases of viral load over 11 days) showed that the 0.1% azelastine group exhibited a greater AUC value of 24.14 ± 13.12 (referring to greater decrease) compared to the placebo group with an AUC value of 18.89 ± 4.70 (*p* = 0.007, Fig. 4). The 0.02% azelastine group showed an AUC value of 22.64 ± 12.56, which was not significantly different from the placebo group (*p* = 0.022, Fig. 4).

Decreases of viral load were also reflected in increases of negative PCR results over time. While PCR results in the placebo group turned negative only on day 11 of treatment, individual patients of the 0.1% azelastine group already showed negative PCR test results from day 2 on. On Day 8, 5 of the 27 (18.5%) and 6 of the 28 (21.4%) patients in the 0.1% azelastine and 0.02% azelastine groups, respectively were negative for the ORF1a/b gene, compared to the 0 of 26 patients in the placebo group. At the end of the treatment, 48.2% of the patients of the 0.1% azelastine group showed no detection of the ORF 1a/b gene, whereas only 23.1% of patients of the placebo group showed negative PCR results (supplementary Table S4).

**Figure 5:**
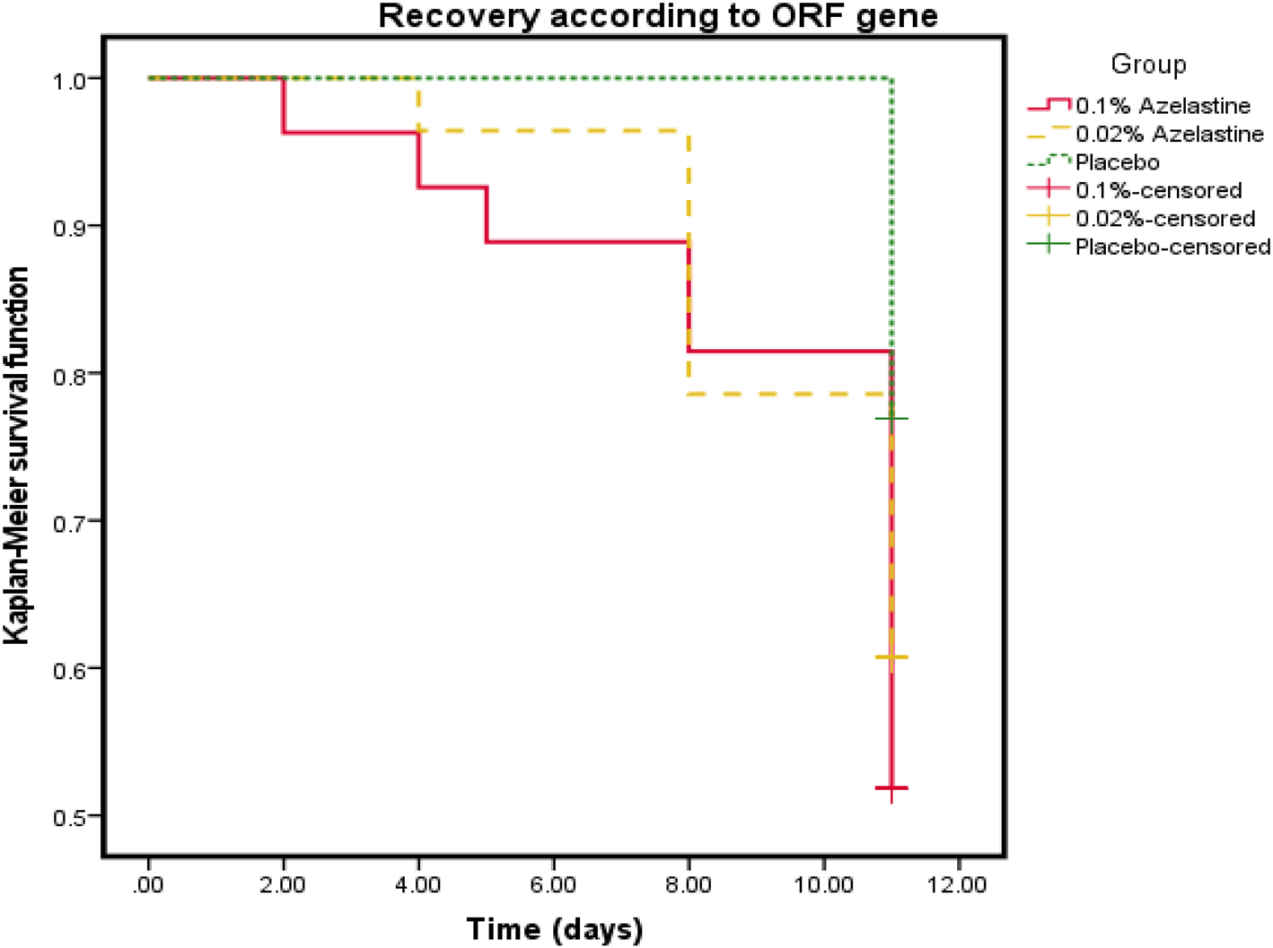
Kaplan–Meier analysis results regarding the ORF 1a/b gene from baseline (day 1) until day 11 of treatment (ITT analysis set).

Kaplan–Meier survival analyses underlined those findings, indicating that mean times of a PCR result to turn negative was 9.96 days (95% CI: 9.02–10.90) in the 0.1% azelastine group, 10.21 days (95% CI: 9.57–10.86) in the 0.02% azelastine group and 11.00 (95% CI: 10.00–10.77) in the placebo group (Fig. 5) Of note, these differences were not statistically significant (*p* = 0.112).

### Development of symptoms

**Figure 6:**
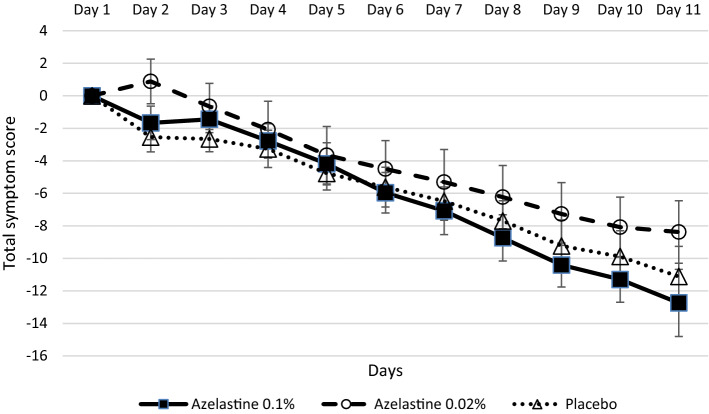
Absolute changes of total symptom scores from baseline (day 1) until day 11 of treatment (ITT analysis set).

The analysis of sum symptom scores showed that the study population (ITT analysis set) suffered from moderate symptoms (mean values ± SD: 38.58 ± 10.04) on day 1 of the study (supplementary Table S5). During the course of the treatment, all study groups showed clear improvements of symptoms (Fig. 6). The azelastine 0.1% azelastine group displayed the greatest improvement of symptoms with 12.74 ± 10.74 mean score reduction. The reduction of the symptom score from baseline to day 11 was 8.38 ± 9.42 in the 0.02% azelastine group and 11.12 ± 9.45 in the placebo group. The reduction in the symptom score was clinically relevant for all three groups.

A closer look at single symptoms confirmed moderate expression of symptoms (supplementary Figure S1) and the general decrease of symptoms over time (supplementary Figure S2). The most common COVID-19 symptoms (loss of sense of smell, loss of taste, fever, cough, and coryza) improved over time in all 3 treatment groups; and no statistical differences were observed between groups. The improvement of the symptom “shortness of breath” was significantly greater on days 3 (*p* = 0.004) and 4 (*p* = 0.011) in the 0.1% azelastine group compared to placebo (supplementary Figure S3).

### Quality of life

The physical and mental health summary scores of the SF-36 questionnaire improved during the course of the treatment without statistical differences between groups (data not shown).

### Patient status (WHO scale)

The WHO clinical progression scale progressively decreased in all treatment groups during the study. At V1, a comparable distribution of patients with a score of 1 (14.8% in the 0.1% azelastine group, 14.3% in the 0.02% azelastine group and 23.1% in the placebo group) or 2 (85.2% in the 0.1% azelastine group, 85.7% in the 0.02% azelastine group and 76.9% in the placebo group) was observed. At the end of the study (day 60), all except one single patient (placebo group) showed a score of 0.

### Body temperature and blood oxygen saturation

Overall, none of the participating patients had clinically relevant increased values of body temperature (data not shown). Similarly, no clinically relevant differences regarding blood oxygen saturation values were detected between groups (data not shown).

### Assessment of overall efficacy and tolerability

59.3% (0.1% azelastine treatment), 50.0% (0.02% azelastine treatment) and 80.8% (placebo treatment) of patients assessed the overall tolerability of the treatment as ‘very good’, which mirrored the tolerability judgement of the investigators, which was assessed as ‘very good’ for 59.3% (0.1% azelastine treatment), 50.0% (0.02% azelastine treatment) and 80.8% (placebo treatment) of patients. The efficacy of the treatment was judged as ‘good’ or ‘very good’ by 75.0% (0.1% azelastine treatment), 74.1% (0.02% azelastine treatment) and 50.0% (placebo treatment) of patients. The investigators judged the efficacy as ‘good’ or ‘very good’ in 74.1% (0.1% azelastine treatment), 82.1% (0.02% azelastine treatment) and 73.1% (placebo treatment) of treated patients. Overall, no statistical differences between groups were determined.

### Safety

The number of possibly and probably related adverse events was comparable between treatment groups (supplementary Table S6), and no safety concerns regarding the treatment regime were raised. Of note, 30 (non-related) adverse events in 13 patients (7 patients with 16 events in the 0.1% azelastine, 2 patients with 4 events in the 0.02% azelastine, and 4 patients with 10 events in the placebo group) were still ongoing at the final safety follow up on day 60. Nineteen of those were common COVID-19 symptoms (shortness of breath [*n* = 4], loss of smell [*n* = 4], loss of taste [*n* = 3], [muscle] weakness [*n* = 2], tiredness/exhaustion [*n* = 2], muscle ache, concentration impaired, headache, and cough).

## Discussion

SARS-CoV-2 infection progression starts with viral entrance mediated by the spike glycoprotein’s interaction with the host ACE2 receptor molecule. Following translocation from nucleus to the endoplasmic reticulum (ER), the sigma-1 receptor (among other factors) plays a role in viral replication. It has been suggested that azelastine can inhibit the entry of the SARS-CoV-2 into the nasal mucosa by binding to the ACE2 receptor and also act via binding to the main protease of SARS-CoV-2 and to the host cell’s sigma-1 receptor, therewith facilitating both viral entry and replication-inhibiting effects^6,9^.

The current proof-of-concept study served to investigate if nasally applied azelastine may have the potential to reduce the viral load (via blocking viral entry and viral replication) in patients tested positively for SARS-CoV-2.

Since the start of the COVID-19 pandemic, its treatment via the nasal route has been studied for a range of drugs^17^. Thus, a nitric oxide nasal spray was shown to reduce the viral load in adult patients with mild COVID-19 infection, and an accelerated SARS-CoV-2 clearance compared to placebo was demonstrated^18^. The preventive application of a hydroxypropyl methyl cellulose nasal spray showed promising results in an observational survey, indicating that it may reduce SARS-CoV-2 infection rates^19^. Treatment of COVID-19 with a hypertonic solution containing seawater, xylitol, panthenol and lactic acid was shown to reduce the viral shedding time in patients with asymptomatic or mild COVID-19^20^, whereas application of povidone iodine nasal spray showed only poor influence on SARS-CoV-2 viral titres^21,22^.

The availability of a self-administrable nasal spray reducing subsequent viral transmission would have great impacts for the community as correlations between SARS-CoV-2 viral load and infectiousness have been shown^23^. In this context, it is interesting to note that publications indicate that individuals vaccinated against SARS-CoV-2 have lower viral loads and are less contagious^24,25^.

Our study population was characterized by an initial mean viral load of log_10_ 6.85 ± 1.31 cp/mL, which was higher than more recently reported SARS-CoV-2 viral load values^26^. The higher viral load value may be explained with the dominance of the alpha (B.1.1.7) SARS-CoV-2 variant during the enrolment phase (Spring 2021, Germany^16^), which is known to infect the human nasal mucosa more efficiently than the wild-type and has been associated with higher viral load^13,14^. Indeed, the majority of the study subjects carried this variant. Whether the current data can be extrapolated to other SARS-CoV-2 variants needs to be investigated. Within this context it is important to point out that in vitro data indicate efficacy of azelastine against various SARS-CoV-2 variants tested^10^.

Upon treatment, a gradual decline of viral load from baseline (day 1) to day 11 of treatment was observed in all three study groups. This is similar to the natural SARS-CoV-2 clearance time of approximately 2 weeks. However, examples of prolonged nasal positivity have also been reported, and many factors are known to have an influence on the individual viral load and clearance^27^.

Importantly, the AUC analysis depicting the viral load decrease based on the detection of the ORF 1a/b gene over the 11-day treatment period showed a significantly greater reduction of virus load in the 0.1% azelastine group compared to placebo. Bearing in mind that viral load might be a surrogate measure of infectiousness, those results are encouraging as they indicate that azelastine may be a promising candidate for preventing the spread of this disease.

Interestingly, significantly greater decrease in viral load was shown on day 4 of treatment in patients with high viral burden (Ct < 25) treated with 0.1% azelastine compared to placebo, indicating that azelastine treatment may be advantageous for this patient population, particularly at an early timepoint of infection. Recent publications indicating that in vitro infectivity correlates with high virus concentrations (Ct ≤ 25) in nasal swabs^28–30^ underline the importance of analysis of this subset population. It would be desirable to extend the investigation of azelastine nasal spray as potential antiviral treatment with in vitro culture experiments.

Of note, we cannot rule out the possibility that the placebo (nasal spray buffer) contributed to viral clearance. In a study examining the effect of azelastine nasal spray on upper respiratory infections in children, it was found that the placebo group, receiving hypertonic saline solution (twice daily) also produced a favourable response compared to those receiving no treatment^31^. Recently, Shmuel et al. reported that a low pH hypromellose nasal powder spray containing common components of nasal sprays could reduce SARS-CoV-2 infection rates^19^. However, a rinsing and diluting effect of the placebo formulation would have led to an underestimation of the effect of the use of the azelastine nasal spray.

The current study demonstrated a gradual decrease of patients’ symptoms and improvements of quality of life. Although no significant differences between groups regarding the total symptom score was shown, it may be speculated that the 0.1% azelastine spray may have positive influences on single symptoms such as “shortness of breath”, which was improved significantly greater in this treatment group compared to placebo at early time points of infection. It would be desirable to study azelastine treatment in a greater COVID-19 population to get further insights on azelastine’s effects on individual symptoms and to determine its potential on long-term symptoms. Quality of life was assessed with the SF-36 questionnaire as no COVID-19 specific patient-reported outcome measures were available at the time of study. We are aware that this limited the capture of COVID-19 specific issues as questions were not specifically aimed for COVID-19 patients. It would be desirable to use a validated, COVID-19 specific questionnaire in future studies, and first attempts for its development are promising^32^.

Patients of the current trial were eligible upon positive PCR test results, and if enrolled no later than 48 h after swab sampling. Thus, it should be kept in mind that treatment started at a time point where the peak of viral load had probably passed. Although it may be expected that the azelastine might be most efficacious during very early time points after infection, its application in the current study setting could only be started during the symptomatic phase of the disease. Importantly, this scenario corresponds to current COVID-19 treatment regimens (e.g., with monoclonal antibodies or antiviral substances), which are usually started at ≤ 5–7 days upon start of symptoms but are still efficacious. Thus, antibody therapy (bamlanivimab and etesevimab) in positively tested, non-hospitalized patients demonstrated that treatment resulted in decreased SARS-CoV-2 viral load by log_10_ − 0.57 on day 11, which was significantly greater compared to placebo (*p* = 0.01)^33^. Comparably, differences in reduction of log_10_ viral load (cp/mL) in our study were − 0.63 (ORF 1a/b gene) comparing treatment with 0.1% azelastine to placebo.

Importantly, newly emerging virus variants have the potential to evade the immune response, thereby affecting the efficacy of specific therapies and underlining the importance of new treatment strategies. This is exemplified by the emergence of the highly immune evasive omicron variant that is resistant to many monoclonal antibodies authorized for clinical use^34^.

Generally, treatment with azelastine appeared safe in SARS-CoV-2 positive patients: no serious adverse events were reported in the current study, and the number of adverse events was comparable between groups. Of note, the known bitter taste of azelastine was only negatively reported by a single patient, and compliance between treatment groups was comparable (mean ± SD: 97 0.12 ± 9.7% compliance), thus indicating that the taste did not negatively influence treatment adherence.

Our study showed both strengths and limitations. Thus, eligibility criteria were designed carefully to investigate a clearly defined, homogeneous study population of low-risk patients with a narrow age range. In addition, intervals between swab sampling were short and the overall number of performed PCR tests was high to allow a very close determination of the viral clearance. However, the overall small number of participants limits conclusions, and results should be interpreted with care. Of note, the mean viral load value showed small variability, thereby supporting the power of the current study.

Overall, the current results are encouraging; however, further studies should be carried out to strengthen the findings, and treatment should be extended to other age and risk groups and cover individuals with different levels of symptom severity.

Of note, pharmacometric analyses of our data indicate that more frequent applications of the nasal spray may be more appropriate for efficient treatment^35^.

Bearing in mind the low number of participants in the current proof-of-concept study, the results still build a promising foundation for a currently running phase III study, during which effects of azelastine nasal spray on symptom severity and progression to severe COVID-19 disease are investigated in a greater patient population.

## Conclusion

Our study results provide the first human data showing that azelastine hydrochloride nasal spray used in a 0.1% concentration may be effective in accelerating the reduction of virus load in the nasal cavity and improving symptoms reported by COVID-19 patients. Future studies will help understanding the impact of azelastine hydrochloride in treating SARS-CoV-2 infected patients.

## Supplementary Information

Supplementary Information 1.

## Data Availability

The data that support the findings of this study are available from URSAPHARM Arzneimittel GmbH but restrictions apply to the availability of these data, which were used under license for the current study, and so are not publicly available. The trial protocol and the data are however available from the authors upon reasonable request and with permission of URSAPHARM Arzneimittel GmbH.
