# Supplementary material for: Early intervention with azelastine nasal spray may reduce viral load in SARS-CoV-2 infected patients
Source: Sci Rep. 2023 Apr 26;13:6839. doi: 10.1038/s41598-023-32546-z (PMC10132439; doi:10.1038/s41598-023-32546-z)
Supplement: Supplementary file 1 — Supplementary Information 1. [file 41598_2023_32546_MOESM1_ESM.docx]

**Supplementary TABLES**

**Table S1**

Table S1: Main characteristics of the treatment groups (safety analysis set). 0·1%: 0·1% azelastine group, 0·02%: 0·02% azelastine group, placebo: placebo group. bmi= body mass index.

|  | | | **Group** | | | |  |
| --- | --- | --- | --- | --- | --- | --- | --- |
|  |  |  | **0·1%** | **0·02%** | **Placebo** | **Total** | **p** |
| Sex | Male | n | 14 | 15 | 15 | 44 | 0·989 |
|  |  | % | 48·3% | 48·4% | 50·0% | 48·9% |  |
|  | Female | n | 15 | 16 | 15 | 46 |  |
|  |  | % | 51·7% | 51·6% | 50·0% | 51·1% |  |
| Age | Mean | | 37·66 | 33·81 | 35·67 | 35·67 | 0·190 |
|  | n | | 29 | 31 | 30 | 90 |  |
|  | Valid n | | 29 | 31 | 30 | 90 |  |
|  | SD | | 12·96 | 12·90 | 13·12 | 12·94 |  |
|  | SEM | | 2·41 | 2·32 | 2·40 | 1·36 |  |
|  | Min | | 19·00 | 19·00 | 19·00 | 19·00 |  |
|  | Median | | 37·00 | 30·00 | 33·50 | 33·00 |  |
|  | Max | | 60·00 | 57·00 | 59·00 | 60·00 |  |
| bmi | Mean | | 25·25 | 25·83 | 23·62 | 24·91 | 0·190 |
|  | n | | 29 | 31 | 30 | 90 |  |
|  | Valid n | | 29 | 31 | 30 | 90 |  |
|  | SD | | 4·95 | 6·04 | 4·58 | 5·27 |  |
|  | SEM | | 0·92 | 1·08 | 0·84 | 0·56 |  |
|  | Min | | 18·17 | 19·16 | 16·90 | 16·90 |  |
|  | Median | | 25·03 | 24·46 | 23·48 | 24·15 |  |
|  | Max | | 44·47 | 44·08 | 42·52 | 44·47 |  |

**Table S2**

Table S2: Baseline (day 1) adjusted decreases in viral load (log_10_ cp/mL) of the ORF 1a/b (ITT analysis set) over the course of the treatment phase. 0·1%: 0·1% azelastine group, 0·02%: 0·02% azelastine group, Placebo: placebo group. * Kruskal Wallis test. **Pairwise comparison performed by Mann Whitney U test (p<0·0167 based on Bonferroni correction).

| **Day** |  | **Baseline adjusted decreases in viral load  (log_10_ cp/mL) of the ORF 1a/b gene** | | | **p** | | | |
| --- | --- | --- | --- | --- | --- | --- | --- | --- |
|  |  | **0·1% (n=27)** | **0·02%**  **(n=28)** | **Placebo**  **(n=26)** | **Overall*** | **0·1% vs 0·02%**** | **0·1% vs Placebo**** | **0·02% vs Placebo**** |
| 2 | Mean (SD) | -0·60 (1·00) | -0·35 (1·12) | -0·38 (1·12) | 0·254 | 0·662 | 0·057 | 0·467 |
|  | Min | -4·13 | -1·99 | -5·17 |  |  |  |  |
|  | Median | -0·61 | -0·46 | -0·29 |  |  |  |  |
|  | Max | 1·45 | 2·50 | 0·90 |  |  |  |  |
| 3 | Mean (SD) | -0·87 (1·22) | -0·68 (1·55) | -0·67 (0·59) | 0·758 | 0·649 | 0·477 | 0·729 |
|  | Min | -4·13 | -5·73 | -1·57 |  |  |  |  |
|  | Median | -0·95 | -0·87 | -0·72 |  |  |  |  |
|  | Max | 2·33 | 3·80 | 0·56 |  |  |  |  |
| 4 | Mean (SD) | -1·67 (1·59) | -1·00 (1·55) | -1·11 (0·65) | 0·095 | 0·138 | 0·027 | 0·782 |
|  | Min | -4·77 | -4·30 | -2·60 |  |  |  |  |
|  | Median | -1·80 | -1·21 | -1·25 |  |  |  |  |
|  | Max | 2·35 | 3·51 | -0·06 |  |  |  |  |
| 5 | Mean (SD) | -2·03 (1·70) | -1·79 (1·63) | -1·67 (0·97) | 0·500 | 0·449 | 0·240 | 0·716 |
|  | Min | -6·82 | -5·73 | -4·79 |  |  |  |  |
|  | Median | -2·01 | -1·63 | -1·57 |  |  |  |  |
|  | Max | 2·37 | 2·54 | -0·08 |  |  |  |  |
| 8 | Mean (SD) | -3·42 (1·90) | -3·62 (2·20) | -2·55 (0·95) | 0·054 | 0·946 | 0·019 | 0·066 |
|  | Min | -6·82 | -7·73 | -4·02 |  |  |  |  |
|  | Median | -3·69 | -2·97 | -2·66 |  |  |  |  |
|  | Max | 0·52 | 1·36 | -0·69 |  |  |  |  |
| 11 | Mean (SD) | -4·45 (2·26) | -4·12 (2·01) | -3·82 (1·61) | 0·406 | 0·556 | 0·188 | 0·426 |
|  | Min | -8·45 | -7·73 | -7·67 |  |  |  |  |
|  | Median | -4·44 | -4·02 | -3·79 |  |  |  |  |
|  | Max | -0·12 | 1·17 | -0·88 |  |  |  |  |

**Table S3**

Table S3: Baseline (day 1) adjusted decreases in viral load (log_10_ cp/mL) of the E gene (ITT analysis set) over the course of the treatment phase. 0·1%: 0·1% azelastine group, 0·02%: 0·02% azelastine group, Placebo: placebo group. * Kruskal Wallis test. **Pairwise comparison performed by Mann Whitney U test (p<0·0167 based on Bonferroni correction).

| **Day** |  | **Baseline adjusted decreases in viral load  (log_10_ cp/mL) of the E gene** | | | **p** | | | |
| --- | --- | --- | --- | --- | --- | --- | --- | --- |
|  |  | **0·1% (n=27)** | **0·02% (n=28)** | **Placebo (n=26)** | **Overall*** | **0·1% vs 0·02%**** | **0·1% vs Placebo**** | **0·02% vs Placebo**** |
| 2 | Mean | -0·63 (0·95) | -0·49 (1·45) | -0·45 (1·09) | 0·334 | 0·533 | 0·088 | 0·678 |
|  | Min | -3·70 | -4·64 | -5·03 |  |  |  |  |
|  | Median | -0·68 | -0·42 | -0·29 |  |  |  |  |
|  | Max | 1·44 | 2·74 | 0·90 |  |  |  |  |
| 3 | Mean | -1·02 (1·40) | -0·69 (1·60) | -0·78 (0·68) | 0·861 | 0·637 | 0·644 | 0·904 |
|  | Min | -4·77 | -5·78 | -1·73 |  |  |  |  |
|  | Median | -1·06 | -0·94 | -0·79 |  |  |  |  |
|  | Max | 2·35 | 4·02 | 0·58 |  |  |  |  |
| 4 | Mean | -2·04 (2·15) | -1·26 (1·97) | -1·67 (1·47) | 0·243 | 0·143 | 0·155 | 0·795 |
|  | Min | -8·27 | -5·78 | -5·42 |  |  |  |  |
|  | Median | -1·89 | -1·36 | -1·47 |  |  |  |  |
|  | Max | 2·34 | 3·91 | -0·08 |  |  |  |  |
| 5 | Mean | -2·17 (1·70) | -2·33 (2·17) | -2·10 (1·45) | 0·778 | 0·987 | 0·522 | 0·556 |
|  | Min | -6·86 | -6·95 | -6·16 |  |  |  |  |
|  | Median | -2·10 | -2·16 | -1·77 |  |  |  |  |
|  | Max | 2·37 | 2·86 | 0·06 |  |  |  |  |
| 8 | Mean | -3·88 (1·79) | -4·42 (2·32) | -3·91 (1·73) | 0·529 | 0·449 | 0·477 | 0·341 |
|  | Min | -6·86 | -8·07 | -7·79 |  |  |  |  |
|  | Median | -3·99 | -3·98 | -3·56 |  |  |  |  |
|  | Max | 0·32 | 1·57 | -1·50 |  |  |  |  |
| 11 | Mean | -5·66 (2·29) | -5·61 (2·29) | -5·47 (2·00) | 0·858 | 0·893 | 0·618 | 0·659 |
|  | Min | -8·75 | -8·78 | -8·12 |  |  |  |  |
|  | Median | -5·48 | -5·81 | -5·18 |  |  |  |  |
|  | Max | -0·86 | 1·34 | -2·01 |  |  |  |  |

Table S4

Table S4: Absolute numbers and cumulative proportions of patients with negative PCR results from day 2 until day 11 as verified by missing detection of the ORF1a/b and the E gene (ITT analysis set). 0·1%: 0·1% azelastine group, 0·02%: 0·02% azelastine group, Placebo: placebo group.

| **Group** | | **0·1%** | | | | **0·02%** | | | | **Placebo** | | | |
| --- | --- | --- | --- | --- | --- | --- | --- | --- | --- | --- | --- | --- | --- |
|  | **Day** | **n** | **recovered patients (n)** | **recovered patients (%)** | **PCR negative patients (cumulative %)** | **n** | **recovered patients (n)** | **recovered patients (%)** | **PCR negative patients (cumulative %)** | **n** | **recovered patients (n)** | **recovered patients (%)** | **PCR negative patients (cumulative %)** |
| ORF 1a/b gene | 1 | 27 | 0 | 0·000 | 0·00% | 28 | 0 | 0·000 | 0·00% | 26 | 0 | 0·000 | 0·00% |
|  | 2 | 27 | 1 | 0·037 | 3·70% | 28 | 0 | 0·000 | 0·00% | 26 | 0 | 0·000 | 0·00% |
|  | 3 | 26 | 0 | 0·000 | 3·70% | 28 | 0 | 0·000 | 0·00% | 26 | 0 | 0·000 | 0·00% |
|  | 4 | 26 | 1 | 0·038 | 7·41% | 28 | 1 | 0·036 | 3·57% | 26 | 0 | 0·000 | 0·00% |
|  | 5 | 25 | 1 | 0·040 | 11·11% | 27 | 0 | 0·000 | 3·57% | 26 | 0 | 0·000 | 0·00% |
|  | 8 | 24 | 2 | 0·083 | 18·52% | 27 | 5 | 0·185 | 21·43% | 26 | 0 | 0·000 | 0·00% |
|  | 11 | 22 | 8 | 0·364 | 48·15% | 22 | 5 | 0·227 | 39·29% | 26 | 6 | 0·231 | 23·08% |
| E gene | 1 | 27 | 0 | 0·000 | 0·00% | 28 | 0 | 0·000 | 0·00% | 26 | 0 | 0·000 | 0·00% |
|  | 2 | 27 | 1 | 0·037 | 3·70% | 28 | 0 | 0·000 | 0·00% | 26 | 0 | 0·000 | 0·00% |
|  | 3 | 26 | 0 | 0·000 | 3·70% | 28 | 0 | 0·000 | 0·00% | 26 | 0 | 0·000 | 0·00% |
|  | 4 | 26 | 0 | 0·000 | 3·70% | 28 | 0 | 0·000 | 0·00% | 26 | 0 | 0·000 | 0·00% |
|  | 5 | 26 | 1 | 0·038 | 7·41% | 28 | 1 | 0·036 | 3·57% | 26 | 0 | 0·000 | 0·00% |
|  | 8 | 25 | 1 | 0·040 | 11·11% | 27 | 2 | 0·074 | 10·71% | 26 | 0 | 0·000 | 0·00% |
|  | 11 | 24 | 5 | 0·208 | 29·63% | 25 | 5 | 0·200 | 28·57% | 26 | 3 | 0·115 | 11·54% |

**Table S5**

Table S5: Absolute values of sum symptom scores over the course of the treatment phase. 0·1%: 0·1% azelastine group, 0·02%: 0·02% azelastine group, Placebo: placebo group, d: day; * Kruskal Wallis test

**Pairwise comparison performed by Mann Whitney U test (p<0·0167 based on Bonferroni correction). ^a^ n=26 on day 6; ^b^ n=24 on day 11, n=26 on days 1 and 9, n=27 on day 8; ^c^ n=25 on day 7; ^d^ n=79 on day 1 and 9, n= 80 on day 6, 7 and 8, n=77 on day 11

| **Day** | **Sum score** | **Group** | | | | **p** | | | |
| --- | --- | --- | --- | --- | --- | --- | --- | --- | --- |
|  |  | **0·1%**  **(n=27)^a^** | **0·02% (n=28)^b^** | **Placebo (n=26)^c^** | **Total (n=81)** | **Overall*** | **0·1% vs 0·02%**** | **0·1% vs Placebo**** | **0·02% vs Placebo**** |
| 1 | Mean (SD) | 41·11 (9·89) | 37·46 (8·93) | 37·08 (11·05) | 38·58 (10·04) | 0·220 | 0·128 | 0·147 | 0·721 |
|  | Min | 23·00 | 23·00 | 21·00 | 21·00 |  |  |  |  |
|  | Median | 41·00 | 34·50 | 36·00 | 37·00 |  |  |  |  |
|  | Max | 61·00 | 57·00 | 60·00 | 61·00 |  |  |  |  |
| 2 | Mean | 39·44 (10·36) | 37·29 (9·22) | 34·54 (12·32) | 37·12 (10·72) | 0·191 | 0·619 | 0·089 | 0·174 |
|  | Min | 23·00 | 11·00 | 11·00 | 11·00 |  |  |  |  |
|  | Median | 39·00 | 36·50 | 33·00 | 36·00 |  |  |  |  |
|  | Max | 63·00 | 60·00 | 59·00 | 63·00 |  |  |  |  |
| 3 | Mean | 39·67 (11·09) | 35·64 (9·93) | 34·42 (12·04) | 36·59 (11·12) | 0·146 | 0·235 | 0·065 | 0·319 |
|  | Min | 23·00 | 15·00 | 21·00 | 15·00 |  |  |  |  |
|  | Median | 40·00 | 34·00 | 29·00 | 34·00 |  |  |  |  |
|  | Max | 64·00 | 62·00 | 61·00 | 64·00 |  |  |  |  |
| 4 | Mean | 38·33 (10·51) | 35·00 (9·22) | 33·81 (11·89) | 35·73 (10·60) | 0·168 | 0·252 | 0·076 | 0·332 |
|  | Min | 22·00 | 21·00 | 21·00 | 21·00 |  |  |  |  |
|  | Median | 39·00 | 33·00 | 31·00 | 34·00 |  |  |  |  |
|  | Max | 62·00 | 62·00 | 59·00 | 62·00 |  |  |  |  |
| 5 | Mean | 36·93 (10·39) | 33·50 (8·66) | 32·31 (11·39) | 34·26 (10·23) | 0·134 | 0·231 | 0·078 | 0·202 |
|  | Min | 21·00 | 21·00 | 21·00 | 21·00 |  |  |  |  |
|  | Median | 35·00 | 31·50 | 30·00 | 31·00 |  |  |  |  |
|  | Max | 59·00 | 60·00 | 61·00 | 61·00 |  |  |  |  |
| 6 | Mean | 35·31 (11·17) | 31·75 (9·43) | 31·46 (11·30) | 32·81 (10·65) | 0·230 | 0·349 | 0·093 | 0·386 |
|  | Min | 21·00 | 11·00 | 21·00 | 11·00 |  |  |  |  |
|  | Median | 33·00 | 31·00 | 28·50 | 30·50 |  |  |  |  |
|  | Max | 62·00 | 59·00 | 61·00 | 62·00 |  |  |  |  |
| 7 | Mean | 34·04 (12·44) | 31·96 (8·53) | 29·68 (10·95) | 31·95 (10·74) | 0·168 | 0·933 | 0·118 | 0·085 |
|  | Min | 21·00 | 21·00 | 21·00 | 21·00 |  |  |  |  |
|  | Median | 30·00 | 30·50 | 26·00 | 29·00 |  |  |  |  |
|  | Max | 65·00 | 59·00 | 61·00 | 65·00 |  |  |  |  |
| 8 | Mean | 32·37 (11·16) | 31·15 (8·61) | 29·38 (10·88) | 30·99 (10·22) | 0·236 | 0·979 | 0·196 | 0·101 |
|  | Min | 21·00 | 21·00 | 21·00 | 21·00 |  |  |  |  |
|  | Median | 29·00 | 29·00 | 26·00 | 29·00 |  |  |  |  |
|  | Max | 60·00 | 61·00 | 61·00 | 61·00 |  |  |  |  |
| 9 | Mean | 30·70 (11·80) | 30·19 (8·58) | 27·85 (10·16) | 29·59 (10·23) | 0·128 | 0·550 | 0·163 | 0·049 |
|  | Min | 21·00 | 21·00 | 21·00 | 21·00 |  |  |  |  |
|  | Median | 27·00 | 27·00 | 25·00 | 27·00 |  |  |  |  |
|  | Max | 60·00 | 61·00 | 59·00 | 61·00 |  |  |  |  |
| 10 | Mean | 29·81 (11·78) | 29·00 (8·35) | 27·19 (10·30) | 28·69 (10·15) | 0·110 | 0·595 | 0·111 | 0·050 |
|  | Min | 21·00 | 21·00 | 21·00 | 21·00 |  |  |  |  |
|  | Median | 26·00 | 27·50 | 24·00 | 25·00 |  |  |  |  |
|  | Max | 63·00 | 61·00 | 60·00 | 63·00 |  |  |  |  |
| 11 | Mean | 28·37 (13·39) | 28·92 (8·72) | 25·96 (10·27) | 27·73 (10·99) | 0·077 | 0·353 | 0·228 | 0·019 |
|  | Min | 1·00 | 20·00 | 21·00 | 1·00 |  |  |  |  |
|  | Median | 24·00 | 27·00 | 23·00 | 24·00 |  |  |  |  |
|  | Max | 62·00 | 61·00 | 61·00 | 62·00 |  |  |  |  |

Table S6

Table S6: Details and numbers of possibly and probably related adverse events. 0·1%: 0·1% azelastine group, 0·02%: 0·02% azelastine group, Placebo: placebo group.

|  |  |  |  | **0·1%** | **0·02%** | **Placebo** |
| --- | --- | --- | --- | --- | --- | --- |
| **Causal relationship to treatment** | **SOC** | **LLT** | **Code** | **n** | **n** | **n** |
| Possibly related | Cardiac disorders | Tachycardia | 10043071 | 1 | 0 | 0 |
|  | Gastrointestinal disorders | Dry mouth | 10013781 | 0 | 0 | 1 |
|  | Infections and infestations | Common cold | 10010106 | 4 | 3 | 5 |
|  |  | Conjunctivitis | 10010741 | 1 | 0 | 4 |
|  |  | Rhinitis | 10039083 | 0 | 0 | 1 |
|  |  | Sinusitis | 10040753 | 0 | 0 | 2 |
|  | Nervous system disorders | Loss of smell | 10024877 | 2 | 3 | 1 |
|  |  | Loss of taste | 10024878 | 1 | 0 | 0 |
|  |  | Sleepiness | 10041014 | 3 | 5 | 5 |
|  | Respiratory, thoracic and mediastinal disorders | Cough | 10011224 | 0 | 0 | 1 |
|  |  | Epistaxis | 10015090 | 1 | 0 | 1 |
|  |  | Nasal mucosa swelling | 10063455 | 0 | 1 | 0 |
|  |  | Nasal sinus blockage | 10075543 | 1 | 0 | 0 |
|  |  | Nose bleed | 10029792 | 0 | 0 | 1 |
|  |  | Sinus pain | 10040747 | 1 | 0 | 0 |
| Probably related | Nervous system disorders | Taste bitter | 10043127 | 0 | 1 | 0 |
|  | Respiratory, thoracic and mediastinal disorders | Dry nasal mucosa | 10085404 | 1 | 0 | 0 |
|  | **Total** |  |  | **16** | **13** | **22** |

**supplementary figures**

**Figure S1**

Figure S1: Single symptom scores (mean ± SD) during treatment (day 1 to day 11; ITT analysis set)

**Figure S2**

Figure S2: changes of single symptom scores (mean ± SD) from baseline to day 11 (ITT analysis set)

**Figure S3**


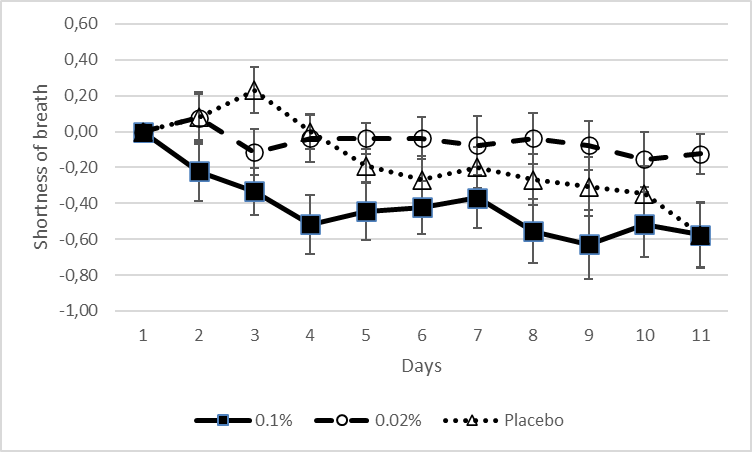


*

*

Figure S3: Development of the symptom “shortness of breath” from day 1 until day 11 of treatment. 0·1%: 0·1% azelastine group, 0·02%: 0·02% azelastine group, placebo: placebo group. * significantly greater improvement on days 3 (p=0.004) and 4 (p=0.011) in the 0·1% azelastine group compared to placebo.

**SUPPLEMENTARY PCR DATA:**

**Testing the potential effect of the placebo nasal spray on the molecular detection of SARS-CoV-2**

To evaluate the potential effect of the placebo solution on the molecular detection (e.g. RNA extraction, reverse transcription or the qPCR) we mixed 100 µl of the viral stock solution (SARS-CoV-2, 3934000 PFU/ml or at 39340 PFU/ml) with equal volume of the placebo solution or with the equal volume of Dulbecco's Modified Eagle Medium (DMEM) as control. After vortexing, RNA was extracted (Monarch Total RNA Miniprep Kit, New England Biolabs) according to the manufacturer’s instruction and the virus was quantified with a reverse transcription real-time PCR specific to the E gene (LightMix Modular SARS-CoV (COVID-19), Cat.No. 53-0776-96 TIB MOLBIOL). For the reaction mix 10 µl of the RNA extract was mixed with 0.5 µl of the reagent mix, 4 µl of Roche master and 0.1 µl RT enzyme and filled to 20 µl with nuclease free PCR grade water. The PCR program is described in the supplementary table S7. For each condition two independent replicates were tested.

**Table S7**

Table S7: PCR Program used for the evaluation of the effect of placebo solution on the molecular detection of SARS-CoV-2.


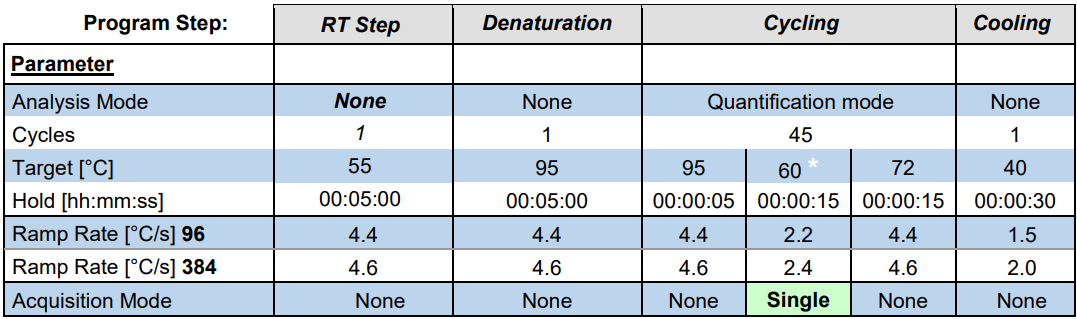


**Results:**

We detected the SARS-CoV-2 E gene by quantifying the RNA extracted from virus stock mixed with the placebo solution or with culture medium (DMEM). The Ct values obtained from two independent replicates indicate no interference of the placebo solution on the molecular detection (see supplementary table S8). At lower viral stock concentration (39340 PFU/ml) the Ct values measured from the placebo mixture were slightly lower than those measured from the cell culture media. While the number of replicates does not allow for statistical comparison, even if this difference was reproducible and significant, the placebo solution would not inhibit the viral detection, rather increase the viral count measured.

**Table S8**

Table S8: Ct values measured from two independent replicates of viral solution mixed with the placebo or with cell culture media.

| **Sample** | **Replicate 1 (Ct-values)** | **Replicate 2 (Ct-values)** |
| --- | --- | --- |
| 393400 PFU SARS-CoV-2 mixed with placebo solution | 10.697 | 11.714 |
| 393400 PFU SARS-CoV-2 isolate mixed with DMEM | 10.257 | 10.276 |
| 3934 PFU SARS-CoV-2 isolate mixed with placebo solution | 18.243 | 18.585 |
| 3934 PFU SARS-CoV-2 isolate mixed with DMEM | 20.205 | 20.399 |
| Placebo solution mixed with DMEM (negative control) | 0 | 0 |
